# Protocol for a single-arm feasibility trial of virtual family-centered rounds: increasing opportunities for family engagement among caregivers with language preference other than English

**DOI:** 10.1186/s40814-025-01599-4

**Published:** 2025-02-12

**Authors:** Adrienne E. Hoyt-Austin, Erika N. Zerda, Daniel J. Tancredi, James P. Marcin, Audriana Ketchersid, Elva T. Horath, Trevor R. Bushong, Daniel S. Merriott, Patrick S. Romano, Kristin R. Hoffman, Jennifer L. Rosenthal

**Affiliations:** 1 Department of Pediatrics, University of California Davis, 2516 Stockton Blvd, Sacramento, CA 95817 USA; 2 Center for Health and Technology, University of California Davis, 4610 X Street, Sacramento, CA 95817 USA; 3 Department of Internal Medicine and Center for Healthcare Policy and Research, University of California Davis, 4150 V St, Sacramento, CA 95817 USA

## Abstract

**Background:**

Telehealth use during family-centered rounds in the neonatal intensive care unit has been shown to shorten length of hospitalization and improve breastfeeding outcomes. For families who speak languages other than English, access to and use of telehealth technologies can be impeded by lack of interpreter services. We aim to evaluate the feasibility of telehealth use during family-centered rounds in the neonatal intensive care unit for families who speak languages other than English.

**Methods:**

In this study proposal, we will conduct an intervention evaluation using the RE-AIM (Reach, Effectiveness, Adoption, Implementation, Maintenance) framework to assess the feasibility of telehealth use during family-centered rounds among families who speak languages other than English in a single-arm feasibility trial. We will provide language-appropriate materials to assist parents with accessing the telehealth technology and bring interpreters into the telehealth encounter directly with neonatal providers. All eligible infants whose families speak languages other than English in a single-site level 4 neonatal intensive care unit during the study period will be included. These families can participate in hospital rounds via telehealth, in-person, or not participate in hospital rounds. We will examine feasibility objectives that assess parental uptake of telehealth for rounds, parental participation in rounds, presence of a certified medical interpreter, telehealth technical issues, and parental survey response rates. We will conduct a mixed methods implementation evaluation using the RE-AIM framework. Exploratory outcomes include parent attendance, length of hospitalization of the infant, human milk feeding, frequency of medical error, parent-reported experience, parental comfort with their child’s care, and parental quality of life will be collected.

**Discussion:**

This study will aid in understanding gaps to telehealth care in languages other than English. We believe this approach will improve health outcomes for hospitalized premature infants, understanding of medical conditions, improve parental quality of life, and reduce inequities in access to healthcare via telehealth technologies.

**Trial registration:**

NCT05917899 Limited English Proficiency Virtual Family-Centered Rounds, first posted June 26, 2023, last update posted November 11, 2024.

## Introduction

Family-centered rounds (FCR) is an essential component of successful pediatric inpatient care that incorporates rounding with the entire clinical team including the parents or guardians (“parents” hereafter) of hospitalized children [1]. For parents, FCR has been shown to reduce anxiety and improve understanding of their child’s medical condition [2, 3]. FCR is associated with improved patient outcomes, reduced exposure to hospital errors, and a shortened length of stay [2]. For critically ill hospitalized infants in the neonatal intensive care unit (NICU), FCR improves parental understanding of the medical conditions of their child and increases shared decision making [4]. Other research has demonstrated that FCR in the NICU is associated with a reduced length of stay and improved breastfeeding outcome [5].

Parental participation in bedside FCR in the NICU can be impeded by limited access to the hospital. NICU care in the United States is increasingly regionalized in large medical centers and many families, especially those living in rural areas, live far from the hospital. Parents can have difficulty attending in person rounds as they may need to return home to care for other children or return to work [6]. Communication and FCR participation for families with language preference other than English (LOE) can be hampered by the need for translation services [7]. Hospitalized children of parents with LOE are more likely to experience adverse events and have worse healthcare outcomes than children of parents with English proficiency [8, 9]. Our group previously demonstrated that the use of videoconferencing with English-speaking families improves engagement in FCR (herein vFCR) and allows families to participate in FCR from home [5].

Despite these promising results, vFCR has potential limitations that require further investigation and resolution. Before broad implementation of vFCR, careful study is needed to prevent the widening of disparities based on language preference and other social factors. Previous work has demonstrated large disparities in access and use of videoconferencing technology within healthcare settings by patient age, gender, race and ethnicity, insurance, and language, whereby families with LOE are half as less likely to have access to pediatric telemedicine care as English-speaking families [10].

### Objectives and trial design

Families with LOE should be prioritized when designing pediatric hospital-based research and implementing evidence-based clinical guidelines. Research in what influences the reach, adoption, and impact of telehealth among diverse groups is critically needed [11, 12]. The Reach, Effectiveness, Adoption, Implementation, Maintenance (RE-AIM) framework is a widely adopted implementation science framework that explicitly focuses on these design and implementation processes [13]. We will use the RE-AIM framework—with the RE-AIM extension to promote sustainability and health equity [14]—to conduct an intervention evaluation that explores the dynamic contextual factors influencing the vFCR intervention implementation for LOE families. In the quantitative phase, we will test the feasibility of vFCR among LOE parents of hospitalized infants in the NICU in a single-arm feasibility trial by providing language-appropriate materials to assist with accessing the vFCR technology and bringing interpreters into the vFCR encounter. In the qualitative phase, we will conduct qualitative interviews with NICU staff and LOE participants about the use of vFCR in the NICU. We will apply the RE-AIM framework [13, 15] to conduct an implementation evaluation within this trial. We will incorporate the RE-AIM extension, which is designed to promote sustainability and health equity [14], by exploring intervention evolvability (adaptability to long-term, dynamic contexts) and by considering equity across all RE-AIM dimensions. We will use a mixed methods approach with a convergent design to merge and categorize the data using the RE-AIM framework [16]. Our research group has reported previously that vFCR is technologically feasible, does not burden the NICU team, and parent-reported outcomes can be collected [5]. As this technology has already been implemented in the study site NICU, the objective of this study is to optimize FCR for LOE families and improve outcomes of hospitalized infants and their families.

## Methods

Reporting of this trial protocol follows the SPIRIT guideline for reporting trial protocols [17]. This study has been registered at clinicaltrials.gov (NCT05917899).

### Setting and population

This study will take place in a 121-bed children’s hospital within a large academic medical center in Northern California, USA. This hospital NICU has 49 beds and accepts patients from over 33 counties from a 65,000-mile region that includes Northern California, Southern Oregon, and Nevada.

Family units will be included in this trial and are defined as the hospitalized infant(s) and their parent(s) or guardian(s). Eligible families will have an infant who is < 365 days of age admitted to the NICU and at least one adult (aged 18 years or older) parent with a preferred language that is other than English. Exclusion criteria are families with restrictions placed by child protective services (such as restricted access to patient information or limited visitation), families previously enrolled in this trial on a prior NICU admission, and/or families already enrolled in a different trial that includes a FCR intervention.

### Study overview

This single-arm feasibility trial will assess the use of vFCR for LOE families with hospitalized infants in the NICU. Over a 12-month study period, we will conduct the quantitative phase and enroll all eligible family units, allowing accurate measurement of denominator values for feasibility objectives. Beginning in the first year and continuing into the second year of the study, we will conduct the qualitative phase and mixed methods evaluation.

We will invite the parents of enrolled family units to subscribe to use vFCR, by providing their contact information (i.e., cell phone number or email) to receive a secure link to join vFCR each weekday morning, excluding university holidays. In this way, subscribed parents have the option to participate in rounds via vFCR with video medical interpreting services or through usual care. Usual care is defined as attending FCR at bedside with a medical interpreter available or no participation in rounds. If family units have more than one parent listed in the electronic health record (EHR), both parents will be separately invited to subscribe to vFCR.

### Participant outreach

A research coordinator will make three attempts to contact each eligible parent until they decline or accept the invitation to subscribe to vFCR. Subsequently, every 2 weeks, new invitations will be sent to those who initially declined participation in vFCR. We will repeat outreach every 14 days because we learned in our previous NICU vFCR pilot trial that sometimes parents decline and then change their decision and would appreciate continued outreach to subscribe to use vFCR throughout their child’s hospitalization [5].

### Intervention

During daily rounds, a computer mounted on a medical grade pole with wheels and an omnidirectional microphone, a speaker, and a video camera will be taken on rounds to conduct the telehealth visits during vFCR. Our study site utilizes Health Insurance Portability and Accountability Act (HIPAA) compliant software called ExtendedCare for telehealth visits; this software launches directly from the patients’ chart in the EHR and integrates with the video medical interpreting platform, Martii. During rounds, the NICU team will launch the application and send a link to the vFCR through ExtendedCare by the participant’s preferred mode of communication (text or email). The link connects the parent directly to vFCR and does not require an additional download or application to use. A video interpreter will be invited to the vFCR when the parent is present. FCR rounds will then proceed with the NICU team. Parents of enrolled participants are not required to participate in vFCR and can join vFCR as frequently as they desire, come to the bedside for in-person FCR, or not participate in rounds at all. If there are technical difficulties, a help desk is available 24 hours per day, 7 days per week for the ExtendedCare application.

### Outcomes

Trial protocol outcome measures are viewable in Table 1.

**Table 1:** vFCR study outcomes

Outcome name	Outcome type	Data source
LOE Parent subscription	Primary feasibility objective	Research team logs, EHR
vFCR use	Feasibility objective	Observation of rounds, Extended Care usage report
Interpreter use during vFCR	Fidelity objective	Observation of rounds, Extended Care usage report for LOE
Technical issues during vFCR	Feasibility objective	Observation of FCR, parent report, helpdesk technical issues log
Data collection	Feasibility objective	Parent surveys
Parent FCR attendance	Exploratory	Observation of rounds
Parent experience	Exploratory	Child HCAHPS survey
Parent activation	Exploratory	P-PAM survey
Parent quality of life	Exploratory	PedsQL Family Impact Module survey
Length of stay, days	Exploratory	EHR
Breast milk feeding	Exploratory	EHR
Adverse events / errors	Exploratory	EHR and solicited reports

*FCR* family-centered rounds, *EHR* electronic health record, *LOE* language preferred other than English, *vFCR* videoconferencing family-centered rounds, *HCAHPS* Child Hospital Consumer Assessment of Healthcare Providers and Systems survey, *P-PAM* Parent-Patient Activation Measure, *PedsQL Family Impact Module* measures parental quality of life and family functioning

### Feasibility objective outcomes

We will test the feasibility of conducting a trial for vFCR in NICU patients with LOE parents. Feasibility will be assessed through the following objectives (Table 2):

1. Objective 1 (primary feasibility objective) will measure the reach of vFCR for LOE families and will be met if at least 75% of enrolled families have at least one parent who subscribes to use vFCR.
2. Objective 2 will monitor uptake of vFCR and will be met if at least 70% of enrolled families use vFCR at least once during their infant’s hospitalization. We will measure the percent of enrolled families that participate in vFCR by tracking logins to the ExtendedCare application that is used for parent-to-provider video access during vFCR.
3. Objective 3 will measure the fidelity the intervention and will be met if at least 95% of vFCR with a parent in attendance whose language preference is other than English includes a professional medical interpreter in the parent’s preferred language.
4. Objective 4 will measure the technical feasibility and will be met if at least 90% of vFCR encounters have no technical issues. This will be assessed using vFCR observations, parent solicited reports, and reviewing the hospital helpdesk technical issues log during the study period.
5. Objective 5 will assess the feasibility of survey data collection with a goal of survey response rates being at least 75% and with missingness for each variable of interest being less than 10%.

**Table 2:** Feasibility objectives

Objectives	Indicator	Criteria for success
*Feasibility* of reach of vFCR	At least one LOE parent subscribes to vFCR	Subscription of at least one parent to vFCR will be at least 75% among LOE-enrolled families
*Feasibility* of uptake of vFCR	Use of vFCR during the trial period	Parent use of vFCR at least once will occur among at least 70% of enrolled families
*Feasibility* of intervention fidelity	Interpreter use for vFCR encounters with a parent in attendance with LOE	Use of a professional interpreter will occur in at least 95% of vFCR encounters that have a LOE parent in attendance
*Feasibility* of technology	Technical issues	Among attempted vFCR encounters, at least 90% will have no technical issues
*Feasibility* of data collection	Survey response for survey-derived outcome	Survey response rate will be at least 75%; all data elements will have less than 10% missing values

*vFCR* virtual family-centered rounds, *LOE* language preferred other than English

### Exploratory outcomes

We will collect exploratory outcomes data to test the feasibility of data collection; this trial is not designed to test hypotheses regarding efficacy. Exploratory outcomes including length of parent attendance of FCR, hospitalization of the infant, human milk feeding, frequency of medical error, parent-reported experience, parental activation (comfort with child’s care), and quality of life will be collected. Parent attendance of FCR will be measured from the date of participation until the date of discharge from the NICU. Parent FCR attendance is defined as the number of FCR attendance days either in person or with videoconferencing (numerator) divided by the infant’s total number of weekday round encounters (denominator).

Length of hospitalization of the infant will be measured as total days in the NICU, based on the midnight census, and will be measured through EHR review. Breastfeeding outcomes are defined as parents’ own human milk feeding and will include breastfeeding initiation or initiation of expression of parental human milk along with any and exclusive parental human milk at NICU discharge. Parental human milk feeding includes feeding directly from the breast/chest or receipt of parental human milk through another mechanism of delivery (such as syringe and bottle). We are defining exclusive human milk feeding as receipt of only parental human milk (with or without medically required fortification) and will be measured through EHR review.

Parent-reported outcomes will be collected via a survey packet. Parent experience will be measured through two items assessing overall experience from the Child Hospital Consumer Assessment of Healthcare Providers and Systems (HCAHPS) Survey [18, 19]. Parent activation, or a parent’s comfort with their child’s care, will be measured using the Parent-Patient Activation Measure (P-PAM) [20, 21]. Parental quality of life will be measured using the PedsQL™ Family Impact Module [22].

Medical errors and adverse events will be obtained via solicited reports and review of EHR data, using procedures as previously described in our prior vFCR pilot study [5].

### Implementation evaluation

#### RE-AIM mixed methods implementation evaluation

We will use the RE-AIM framework to conduct an implementation evaluation within this trial [15, 23]. Mixed methods with a convergent design [24] will be utilized to evaluate the vFCR intervention among LOE families.

#### Quantitative phase

Descriptive statistics will be used to describe the Reach, Effectiveness, Adoption, and Implementation Domains. We will use the existing Extended Care usage report to obtain data on vFCR encounters that use an interpreter during the 3 months after the trial ends (Maintenance). To characterize Reach and Adoption, we will report data by groups defined by infant, parent(s), and family characteristics. Imputation will not be used for missing data.

#### Qualitative phase

In the qualitative phase, we will conduct interviews with parent participant(s) and NICU staff to assess the implementation strategy using an interview guide that incorporates the RE-AIM implementation framework [15, 23]. Convenience and then purposive sampling [25] will be used to recruit parent participants as well as NICU staff (i.e., nurses, physicians, social workers). Parents will include both those who subscribe to use vFCR and those who decline. For parent participants, we will prioritize conducting the interview with a study investigator who is a certified interpreter in the preferred language of the participant family. For example, a Spanish-speaking investigator who is a certified interpreter (EH) will interview a Spanish-speaking family. For families where we do not have a language-congruent investigator who is a certified interpreter, we will utilize an interpreter in the patient’s preferred language for the interview.

The interview guide will explore factors influencing the uptake of vFCR in LOE families. We will include parents of infants who were subscribed and those who declined. Qualitative interviews will be completed with one-on-one interviews that will take approximately 45 min and will be audio recorded, transcribed, and then reviewed for accuracy. We will conduct one-on-one interviews with 30 individuals or until we reach thematic saturation.

We will use an inductive and deductive approach to iteratively examine the qualitative data. A framework analysis of codes selected a priori pertaining to the 5 components of RE-AIM will be used to code the initial three transcripts to allow for the development of emergent codes [15]. Four researchers will use the emergent codes from the initial analysis to code an additional 2–5 transcripts. Researchers will meet at regular intervals to ensure consensus on code use, refining codes, addition of codes, and develop categories and analytic themes. These data will be used to refine the interview guide as needed. Data will be organized in ATLAS.ti software [26]. We will repeat this process every 2–5 transcripts and revisit analyzed transcripts as new codes are identified. The qualitative investigators will meet to refine codes into analytic themes until data saturation is met.

### Integration

We will use a convergent design [16] to integrate the quantitative and qualitative data. We will compare quantitative and qualitative data at qualitative analysis meetings to permit the opportunity to explore emerging findings in subsequent interviews. Data will be merged and categorized into the domains of the RE-AIM framework [15]. These converging data will allow us to refine our approach based on the integration results (see Table 3).

**Table 3:** The use of RE-AIM for quantitative and qualitative phases

Dimension	Quantitative items	Qualitative items
Reach	% excluded and reasons Characteristics of LOE families who subscribe to use vFCR	Explore factors influencing reach Explore factors influencing subscription
Effectiveness	Heterogeneity of exploratory outcomes in subscribers	Explore mechanisms of potential heterogeneity effects Explore unmeasured effects of the intervention
Adoption	Subscriber use of vFCR	Explore factors influencing parent participation Explore factors influencing medical staff participation
Implementation	Use of professional interpreter in vFCR encounter % of vFCR encounters with technical issues	Explore factors impacting the use of medical interpreters Explore factors influencing implementation
Maintenance	Number of vFCR encounters with LOE families during the 3-months after the trial ends	Explore factors influencing sustainability

*vFCR* virtual family-centered rounds, *LOE* language preferred other than English

### Participant timeline

We will conduct the feasibility study over a 12-month period. Participation in the study and the intervention delivery will occur in the first 12 months. The RE-AIM mixed methods evaluation will occur during the first and second year with the completion of data collection, analysis, and integration occurring in the second year.

### Sample size

The sample size of this feasibility study is calculated at *n* = 36. This sample size will allow us to estimate proportions in feasibility objectives 1, 2, and 5 with error margins ≤ 16.3 percentage points. Eligible participants will be approached by a research assistant during daytime hours on weekdays to reach the target sample size. Based on our previous pilot vFCR study at the same study site, we anticipate exceeding the sample size goal and enrolling up to 45 participants during the enrollment period [5].

### Data collection

Data collection will be ongoing and include daily weekday vFCR observations, parent surveys, and reviews of the EHR and technical issues log. Adherence to the feasibility objectives will be monitored. For objective 1, we will use daily chart review to track the number of LOE-eligible infants indicated by preferred language in the infant chart and calculate the percentage subscribed based on this total number. If the preferred language is not listed, research coordinators will cross-reference notes in the medical record for admitted patients in the NICU to determine the language preferences of the family. For objective 2, we will track logging in to the ExtendedCare application for subscribed families to determine the percentage that access the technology. Encounters will be measured using a dichotomous rating among the vFCR encounters with a LOE parent(s) in attendance of whether the FCR encounter includes the presence of the professional medical interpreter for objective 3. Objective 4 will be assessed using FCR observations, parent-solicited reports, and helpdesk technical issues log. Objective 5 will be assessed by survey response rates and by missing data elements for each variable of interest.

Data collection for exploratory outcomes will be collected primarily through several methods. FCR parent attendance is collected by the NICU rounding team during weekday rounds where a data collection sheet is marked if a parent joins FCR. EHR review will be used at discharge to measure length of stay and any human milk feeding. Medical errors and adverse events will be reviewed by comparing EHR data with the study site’s internal incident reporting system along with solicited reports, as previously described in the vFCR pilot study [5].

Exploratory outcomes of parent experience, parental activation, and parent quality of life will be measured at hospitalization discharge through a survey packet. Surveys will be administered to one parent per enrolled family; this parent will be the parent with a language preference other than English. For families with more than one parent with a language preference other than English, the family will select which parent will complete the survey packet. Survey packets will be translated into the six most spoken languages among parents of infants in our NICU (Spanish, Russian, Pashto, Arabic, Farsi, Punjabi). For parents with a preferred language other than these six languages for whom we will not have prepared language-appropriate packets, we will administer the surveys verbally using a professional interpreter. Surveys will be distributed at the time of NICU discharge. Non-respondents will be followed up 7 days after discharge. If surveys are not returned by 21 days post discharge, the survey will be considered a non-response.

### Data management

The vFCR encounter through the ExtendedCare platform is HIPAA compliant and is not recorded. Survey and demographic information collected from participants will be deidentified, collected through REDCap [27], and managed by trained research staff at the study site. Qualitative interviews will be recorded, transcribed, and reviewed by the study team for accuracy. Digital audio recordings, transcriptions, and study notes will be stored on a secure password-protected HIPAA-compliant share drive available to researchers at the study site. Transcribed qualitative interviews will exclude identifiable information to protect participant privacy.

### Statistical methods

For all feasibility objectives outlined above in the “Feasibility Objective Outcomes” section, we will report proportions and exploratory outcome estimates with 95% confidence intervals (CI), consistent with SPIRIT recommendations [17], using Wilson score intervals for proportions. For the FCR caregiver attendance exploratory outcome, to account for variation in the duration of patient hospitalizations and thus the number of FCR encounters, we will report the duration-weighted mean for the FCR caregiver daily attendance proportion using Poisson regression with offset term based on the number of weekdays and with confidence intervals adjusted for clustering at the family-level. Similarly, for the medical error exploratory outcome, we will estimate adjusted rates using Poisson regression with offset term based on the total length of stay.

### Data monitoring

We will not require a data monitoring committee as this is a low-risk intervention. This feasibility study will use mixed methods, where qualitative and quantitative data analysis will occur throughout the trial, as outlined above. Data analysis will be ongoing at each qualitative data meeting, and we will collect, assess, and report on any unexpected harm.

### Ethics and dissemination

This feasibility trial is registered on ClinicalTrials.gov (NCT05917899). The protocol has been approved by the study site Institutional Review Board (IRB) and has been deemed minimal risk (only concern is loss of confidentiality). Participant privacy and confidentiality will be maintained by trained research staff. We will store data in password-protected and encrypted computer software. We will destroy data 7 years after the completion of the study. We will disseminate the results of this study through presentations at national/international scientific conferences and peer-reviewed publications. We will submit all peer-reviewed publications resulting from this feasibility trial to PubMed Central digital archive. After completion of the study, the dataset will be available from the corresponding author on reasonable request. The investigators have no competing financial interests in this feasibility trial.

Informed consent will be obtained at the time of participation for parent-reported survey outcomes and qualitative interviews. This research has a waiver of consent as vFCR is an available clinical resource for all hospitalized patients at the study site. No clinician or parent is required to use the intervention. We will not collect biological specimens. Protocol amendments will be reported to and approved by the study sponsors and the study site IRB as needed.

Any adverse events will be assessed by the study team and principal investigator. If any event is determined to be unanticipated, serious, and possibly related to the study intervention it will be reported to the study site IRB within 10 days. Any adverse events that are determined to be unrelated to the study will be reported to the IRB per internal IRB policy for continuing review.

## Discussion

This feasibility study of the use of vFCR to include families with LOE (a language preference other than English) will aid in understanding gaps to telehealth care in languages other than English. We believe this approach will improve health outcomes for hospitalized premature infants, understanding of medical conditions, and improve parental quality of life. Importantly, we believe that centering families with LOE as we assess our implementation strategy of use of vFCR may be one of many approaches to close inequities in access to healthcare via telehealth technologies [8–10].

## Data Availability

Data will be shared upon reasonable request.

## References

[1] COMMITTEE ON HOSPITAL CARE and INSTITUTE FOR PATIENT- AND FAMILY-CENTERED CARE; Patient- and Family-Centered Care and the Pediatrician's Role. Pediatrics. 2012;129(2):394–404. 10.1542/peds.2011-3084 10.1542/peds.2011-3084 22291118

[2] Forsythe P. New practices in the transitional care center improve outcomes for babies and their families. J Perinatol. 1998;18(6 Pt 2 Su):S13–7. 10023374

[3] Subramony A, Schwartz T, Hametz P. Family-centered rounds and communication about discharge between families and inpatient medical teams. Clin Pediatr (Phila). 2012;51(8):730–8. 10.1177/0009922812446012. 22566708 10.1177/0009922812446012

[4] Penticuff JH, Arheart KL. Effectiveness of an intervention to improve parent-professional collaboration in neonatal intensive care. J Perinat Neonatal Nurs. 2005;19(2):187–202. 10.1097/00005237-200504000-00016. 15923969 10.1097/00005237-200504000-00016

[5] Rosenthal JL, Sauers-Ford HS, Williams J, Ranu J, Tancredi DJ, Hoffman KR. Virtual family-centered rounds in the neonatal intensive care unit: a randomized controlled pilot trial. Acad Pediatr. 2021;21(7):1244–52. 10.1016/j.acap.2021.03.007. 33746043 10.1016/j.acap.2021.03.007 PMC8429071

[6] Greene MM, Rossman B, Patra K, Kratovil A, Khan S, Meier PP. Maternal psychological distress and visitation to the neonatal intensive care unit. Acta Paediatr. 2015;104(7):e306–13. 10.1111/apa.12975. 25684177 10.1111/apa.12975 PMC4792263

[7] Clemans-Cope L, Kenney G. Low income parents’ reports of communication problems with health care providers: effects of language and insurance. Public Health Rep. 2007;122(2):206–16. 10.1177/003335490712200210. 17357363 10.1177/003335490712200210 PMC1820424

[8] Khan A, Yin HS, Brach C, et al. Association between parent comfort with English and adverse events among hospitalized children. 2020;02115(12). 10.1001/jamapediatrics.2020.3215 10.1001/jamapediatrics.2020.3215 PMC7573792 33074313

[9] Eneriz-Wiemer M, Sanders LM, Barr DA, Mendoza FS. Parental limited English proficiency and health outcomes for children with special health care needs: a systematic review. Acad Pediatr. 2014;14(2):128–36. 10.1016/j.acap.2013.10.003. 24602575 10.1016/j.acap.2013.10.003

[10] Rosenthal J, O’Neal C, Sanders A, Fernandez Y Garcia E. Differential use of pediatric video visits by a diverse population during the COVID-19 pandemic: a mixed-methods study. Front Pediatr. 2021;9. 10.3389/FPED.2021.645236 10.3389/fped.2021.645236 PMC8311026 34322458

[11] Anaya YB, Martinez L, Vargas-Bustamante A. Telehealth and COVID-19: policy considerations to improve access to care. 2020.

[12] Telehealth wasn’t designed for non-English speakers - the Verge. Accessed December 4, 2024. https://www.theverge.com/21277936/telehealth-english-systems-disparities-interpreters-online-doctor-appointments

[13] Glasgow RE, Harden SM, Gaglio B, et al. RE-AIM Planning and evaluation framework/: adapting to new science and practice with a 20-year review. 2019;7(March). 10.3389/fpubh.2019.00064 10.3389/fpubh.2019.00064 PMC6450067 30984733

[14] Shelton RC, Chambers DA, Glasgow RE. An extension of RE-AIM to enhance sustainability: addressing dynamic context and promoting health equity over time. Front Public Health. 2020;8:501105. 10.3389/FPUBH.2020.00134/BIBTEX. 10.3389/fpubh.2020.00134 PMC7235159 32478025

[15] Glasgow RE, Vogt TM, Boles SM. Evaluating the public health impact of health promotion interventions: the RE-AIM framework. Am J Public Health. 1999;89(9):1322–7. 10.2105/AJPH.89.9.1322. 10474547 10.2105/ajph.89.9.1322 PMC1508772

[16] Creswell John W, Plano Clark Vicki L. Designing and Conducting Mixed Methods Research. 3rd ed. Thousand Oaks: SAGE Publications; 2017.

[17] Chan AW, Tetzlaff JM, Altman DG, et al. SPIRIT 2013 statement: defining standard protocol items for clinical trials development of the spirit 2013 statement. 2013;158. www.annals.org

[18] Toomey SL, Zaslavsky AM, Elliott MN, et al. The development of a pediatric inpatient experience of care measure: child HCAHPS. Pediatrics. 2015;136(2):360–9. 10.1542/peds.2015-0966. 26195542 10.1542/peds.2015-0966 PMC5036167

[19] Giordano LA, Elliott MN, Goldstein E, Lehrman WG, Spencer PA. Development, implementation, and public reporting of the HCAHPS survey. Med Care Res Rev. 2010;67(1):27–37. 10.1177/1077558709341065. 19638641 10.1177/1077558709341065

[20] Hibbard JH, Mahoney ER, Stockard J, Tusler M. Development and testing of a short form of the patient activation measure. Health Serv Res. 2005;40(6 Pt 1):1918–30. 10.1111/j.1475-6773.2005.00438.x. 16336556 10.1111/j.1475-6773.2005.00438.x PMC1361231

[21] Pennarola BW, Rodday AM, Mayer DK, et al. Factors associated with parental activation in pediatric hematopoietic stem cell transplant. Med Care Res Rev. 2012;69(2):194–214. 10.1177/1077558711431460. 22203645 10.1177/1077558711431460 PMC4160822

[22] Varni JW, Sherman SA, Burwinkle TM, Dickinson PE, Dixon P. The PedsQL™ Family Impact Module: preliminary reliability and validity. Health Qual Life Outcomes. 2004;2:1–6. 10.1186/1477-7525-2-55. 15450120 10.1186/1477-7525-2-55 PMC521692

[23] Glasgow RE, Harden SM, Gaglio B, et al. RE-AIM planning and evaluation framework : adapting to new science and practice with a 20-year review. 2019;7(March). 10.3389/fpubh.2019.00064 10.3389/fpubh.2019.00064 PMC6450067 30984733

[24] Creswell J, Clark V. Designing and Conducting Mixed Methods Research. 3rd ed. SAGE Publications; 2017.

[25] MaDC T. Purposive sampling as a tool for informant selection. Ethnobot Res Appl. 2007;5:147–58.

[26] ATLAS.ti Scientific Software Development GmbH. ATLAS.ti Mac (version 23.2.1) [Qualitative data analysis software]. 2023. https://atlasti.com

[27] Harris PA, Taylor R, Minor BL, et al. The REDCap consortium: Building an international community of software platform partners. J Biomed Inform. 2019;95:103208. 10.1016/j.jbi.2019.103208. 31078660 10.1016/j.jbi.2019.103208 PMC7254481

